# The value of the tumour-stroma ratio for predicting neoadjuvant chemoradiotherapy response in locally advanced rectal cancer: a case control study

**DOI:** 10.1186/s12885-021-08516-x

**Published:** 2021-06-25

**Authors:** Yanting Liang, Yaxi Zhu, Huan Lin, Shenyan Zhang, Suyun Li, Yanqi Huang, Chen Liu, Jinrong Qu, Changhong Liang, Ke Zhao, Zhenhui Li, Zaiyi Liu

**Affiliations:** 1 Guangdong Cardiovascular Institute, Guangdong Provincial People’s Hospital, Guangdong Academy of Medical Sciences, Guangzhou, China; 2 Department of Pathology, The Sixth Affiliated Hospital of Sun Yat-sen University, Guangzhou, China; 3 School of Medicine, South China University of Technology, Guangzhou, China; 4 Department of Radiology, Guangdong Provincial People’s Hospital, Guangdong Academy of Medical Sciences, 106 Zhongshan Er Road, Guangzhou, 510080 China; 5 The Second School of Clinical Medicine, Southern Medical University, Guangzhou, China; 6 Department of Radiology, The Affiliated Cancer Hospital of Zhengzhou University & Henan Cancer Hospital, Zhengzhou, China; 7 Department of Radiology, Yunnan Cancer Center, The Third Affiliated Hospital of Kunming Medical University, Yunnan Cancer Hospital, Kunming, 650118 China

**Keywords:** Whole-slide images, Tumour-stroma ratio, Locally advanced rectal cancer, Neoadjuvant chemoradiotherapy response, Tumour regression grade

## Abstract

**Background:**

The tumour-stroma ratio (TSR) is recognized as a practical prognostic factor in colorectal cancer. However, TSR assessment generally utilizes surgical specimens. This study aims to investigate whether the TSR evaluated from preoperative biopsy specimens by a semi-automatic quantification method can predict the response after neoadjuvant chemoradiotherapy (nCRT) of patients with locally advanced rectal cancer (LARC).

**Methods:**

A total of 248 consecutive patients diagnosed with LARC and treated with nCRT followed by resection were included. Haematoxylin and eosin (HE)-stained sections of biopsy specimens were collected, and the TSR was evaluated by a semi-automatic quantification method and was divided into three categories, using the cut-offs determined in the whole cohort to balance the proportion of patients in each category. The response to nCRT was evaluated on the primary tumour resection specimen by an expert pathologist using the four-tier tumour regression grade (TRG) system.

**Results:**

The TSR can discriminate patients that are major-responders (TRG 0–1) from patients that are non-responders (TRG 2–3). Patients were divided into stroma-low (33.5%), stroma-intermediate (33.9%), and stroma-high (32.7%) groups using 56.3 and 72.8% as the cutoffs. In the stroma-low group, 58 (69.9%) patients were major-responders, and only 39 (48.1%) patients were considered major-responders in the stroma-high group (*P* = 0.018). Multivariate analysis showed that the TSR was the only pre-treatment predictor of response to nCRT (adjusted odds ratio 0.40, 95% confidence interval 0.21–0.76, *P* = 0.002).

**Conclusion:**

An elevated TSR in preoperative biopsy specimens is an independent predictor of nCRT response in LARC. This semi-automatic quantified TSR could be easily translated into routine pathologic assessment due to its reproducibility and reliability.

**Supplementary Information:**

The online version contains supplementary material available at 10.1186/s12885-021-08516-x.

## Introduction

Colorectal cancer (CRC) is one of the common causes of cancer-related death worldwide, and approximately one-third of CRC cases occurs in the rectum [1, 2]. Due to the high risk of locoregional recurrence, neoadjuvant chemoradiotherapy (nCRT) is recommended as the standard therapy for locally advanced rectal cancer (LARC) patients [3]. The addition of nCRT as part of LARC treatment has improved the overall survival (OS) and locoregional relapse-free survival, compared to primary surgery alone. However, due to many factors, including individual differences, tumour size, clinical T and N stages, tumour differentiation, treatment-related factors and so on, patients with LARC show varied responses to nCRT due to individual differences. Nearly 20% of patients show resistance to nCRT, demonstrating minimal regression or even tumour progression [4]. Therefore, it is crucial to predict the therapeutic effect of nCRT for patients before treatment, allowing the selection of LARC patients who would or would not benefit from nCRT, to reduce the immunotoxicity and organ toxicity associated with ineffective nCRT [5, 6], and to choose further treatment methods.

Currently, it has become increasingly known that the composition of the tumour microenvironment as well as tumour-stroma interactions play a major role in tumour progression, metastasis, and therapy resistance, leading to poor clinical outcomes [7]. The stroma is one of the key components in the tumour microenvironment. The stromal compartment secretes growth factors and stimulates the formation of new blood vessels to provide the growing tumour with oxygen and nutrients [8]. Several studies have also suggested that the stroma contains more prognostic information than the tumour epithelial component [9, 10].

Additionally, there have been different studies that found that the tumour-stroma ratio (TSR) is a valid and practical prognostic factor in solid tumours, including oesophageal cancer, endometrial carcinoma, non-small-cell lung cancer, and CRC [11–14]. Most studies have evaluated the TSR on surgical paraffin sections, while only a few studies have evaluated this parameter on biopsy specimens [7, 15]. A retrospective study from Pelt et al. investigated the value of TSR based on biopsy specimen assessment to predict nCRT response in patients with oesophageal cancer [7]. However, a subjective method was used in their study to evaluate the TSR, with disagreement among pathologists. Currently, digital pathology, especially whole-slide imaging (WSI), is increasingly being used in clinical practice, and is very suitable for full quantitative evaluation of the TSR. It is worthwhile to investigate whether fully quantitative determination of the TSR in preoperative biopsy specimens can predict nCRT efficacy in LARC patients.

Therefore, the objective of this study was to explore whether the TSR evaluated by a semi-automatic quantification method in preoperative biopsy specimens can predict nCRT response in LARC patients, thereby reducing unnecessary pain and cost.

## Materials and methods

### Patients

A total of 248 consecutive patients diagnosed with clinical stage I–III LARC who underwent nCRT followed by resection at the Sixth Affiliated Hospital of Sun Yat-sen University (SYSU6) between November 2012 and November 2017 were enrolled in the study. The specific inclusion and exclusion criteria are shown in the supplementary material (e.g. Additional file 1). We collected clinicopathologic data from medical records as follows: age, sex, histopathological type, differentiation grade, pre-treatment clinical T and N status, pre-treatment CEA level, pre-treatment CA199 level, tumour location, time interval between nCRT and surgery, and neoadjuvant radiotherapy dose. All patients received chemoradiotherapy. The combined radiochemotherapy included capecitabine/long-course radiotherapy (45–50 Gy in 25–28 fractions), infusional 5-fluorouracil/long-course radiotherapy (45–50 Gy in 25–28 fractions), or bolus 5-fluorouracil/leucovorin/long-course radiotherapy (45–50 Gy in 25–28 fractions). Mono chemotherapy included FOLFOX (folinic acid, fluorouracil and oxaliplatin) or CAPEOX (capecitabine and oxaliplatin). The mono radiotherapy was the regimen of 25 Gy in 5 fractions. The institutional review board of SYSU6 approved this retrospective study (approval no. 2019ZSLYEC-169 date: 2019-06-12), and informed consent was waived.

### Tissue slide preparation and scanning

For each patient, formalin-fixed paraffin-embedded sections of biopsy specimens were cut to a thickness of 4 μm and stained with haematoxylin and eosin (HE). These slides were scanned by digital whole-slide scanning (Leica, Aperio-AT2, USA) at 40× magnification.

### Semi-automatic computation of the TSR

**Fig. 1:**
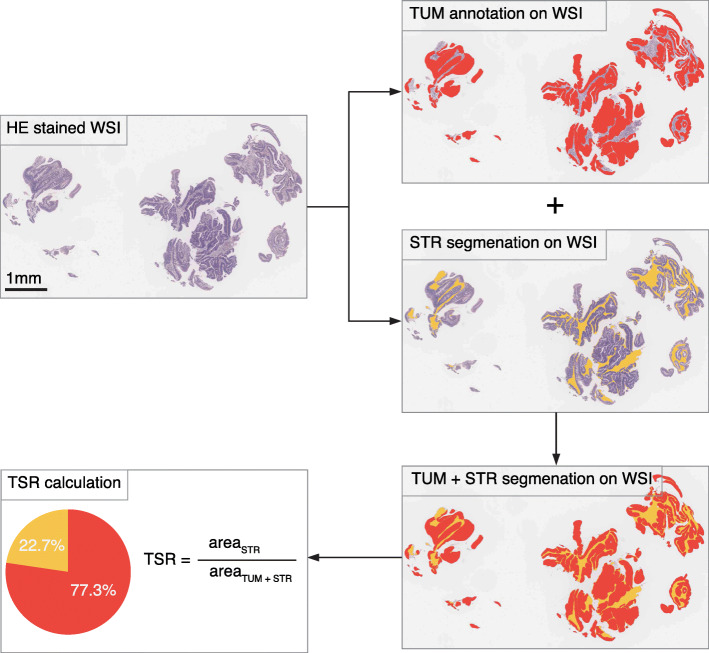
Semi-automatic tumour-stroma ratio computation workflow. An expert pathologist annotated the tumour epithelium and the whole tumour region (including the tumour epithelium and stroma) on the WSI. The tumour epithelium region is marked in red, and the stroma region is marked in yellow. The stromal and tumour epithelial areas were calculated from the segmentation map. The TSR was defined as the stroma proportion in the sum of the stroma and tumour epithelial areas. TSR, tumour-stroma ratio; WSI, whole-slide image

An expert pathologist (more than 10 years of experience) annotated the tumour epithelium and the whole tumour region (including the tumour epithelium and stroma) on WSIs via ImageScope (version 12.4.3, Leica, USA). To find the stromal region automatically, the following image processing was performed in the MATLAB environment (R2019a, MathWorks, USA): The original image (40×, 0.250 μm/pixel) was scaled to a smaller image (10×, 1.00 μm/pixel). Then the scaled colour image (RGB-encoded) was converted into a greyscale image, and Gaussian smoothing with a standard deviation of 15 was added. Otsu’s method was used to determine the global threshold, segmenting the grey image into the background and tissue region mask (mask_TIS_). As we already have the annotated tumour epithelium (mask_TUM_) and the whole tumour (mask_WHO_TUM_) regions, the stroma region (mask_STR_) was calculated by Boolean operation: mask_STR_ = (mask_TUM_ & mask_TIS_) xor (mask_WHO_TUM_ & mask_TIS_). The calculation process was present in Fig. 1.

The TSR was defined as follows: TSR = stromal area / (tumour epithelial area + stromal area) × 100%. A value of 50% was used to categorize patients into two groups: stroma-high and stroma-low, as determined in previous studies to be the most discriminative [16]. To more accurately stratify patients and develop suitable treatment options for individualized precision therapy, we also adopted a 3-category TSR classification which divided patients into stroma-low, stroma-intermediate, and stroma-high groups, using the cutoffs determined from the whole cohort to balance the proportion of patients in each category. The cutoffs were not defined based on the ROC curve, thus avoiding overfitting of the data.

Since a WSI often takes a few hours to be annotated, another pathologist re-annotated 30 image blocks (0.5 mm × 0.5 mm) from ten randomly selected WSIs to assess inter-observer variability of the annotation. The selected regions should contain only tumour epithelial and tumour stroma and exclude necrotic or inflammatory regions. The intra-class correlation coefficient (ICC) with 95% confidence interval (CI) was calculated.

### Evaluation of the response to nCRT

Tumour regression grade (TRG) is significantly associated with disease recurrence and patient survival and can assess the response to nCRT [17]. Therefore we evaluated the response to nCRT by an expert pathologist using the four-tier TRG system defined by the American Joint Committee on Cancer (AJCC, eighth edition) [18]. The pathologist compared the biopsy and the tumour resection specimen to derive the final TRG. TRG 0 is defined as complete regression with no visible cancer cells and is called a pathological complete response. TRG 1 is characterized by single or small groups of tumour cells. TRG 2 is characterized by residual cancer outgrown by fibrosis. TRG 3 is defined by minimal or no tumour cells killed. The TRG scores were confirmed by pathologic examination after surgical treatment. We categorized the treatment response into two groups. Responses of TRG 0–1 were categorized as major-responders, whereas TRG 2–3 were classified as non-responders.

### Statistics

According to the rule of thumb [19], a minimum of 10 events per variable were necessary for the multivariate analysis. The sample size in our multivariate analysis was sufficient (*n* = 248). All data analyses were conducted in R software (version 3.6.1). Continuous factors, such as age, were analysed with t tests, while categorical characteristics were tested with Pearson chi-square test or Fisher’s exact test. Inter-observer variability was assessed by ICC analysis. The logistic regression model was used to perform univariate and multivariable analyses. Parameters associated with treatment response in univariate analysis were selected for multivariable analysis. In all analyses, a two-tailed *P* value < 0.05 was regarded as statistically significant.

## Results

### Patient and tumour characteristics

A total of 248 LARC patients were recruited in this study. The median age was 55 (range, 25 to 79) years, and 176 (71%) were males. Two hundred thirty-two (93.5%) tumours were moderately or well differentiated, and 16 (6.5%) tumours were poorly differentiated. Table 1 lists the demographics and clinicopathological parameters. The TRG results for patients treated with nCRT were as follows: 27.0% TRG 0 (*N* = 67); 31.9% TRG 1 (*N* = 79); 37.9% TRG 2 (*N* = 94); and 3.2% TRG 3 (*N* = 8). These results are consistent with most other studies [20, 21]. After dichotomization, 146 patients were categorized as major-responders (TRG 0–1), whereas 102 patients were classified as non-responders (TRG 2–3).

**Table 1:** Characteristics of patients in the whole cohort

	Total	Major-responders	Non-responders	***P***
	***N*** = 248	***N*** = 102	***N*** = 146	
**Age (year, mean ± SD)**	54.61 ± 11.61	54.20 ± 11.17	55.20 ± 12.25	0.514
**Sex**				0.548
Male	176 (71.0%)	101 (69.2%)	75 (73.5%)	
Female	72 (29.0%)	45 (30.8%)	27 (26.5%)	
**Histopathological type**				0.793
Adenocarcinoma	236 (95.2%)	138 (94.5%)	98 (96.1%)	
Mucinous adenocarcinoma	12 (4.8%)	8 (5.5%)	4 (3.9%)	
**Differentiation grade**				0.274
Well or moderate	232 (93.5%)	134 (91.8%)	98 (96.1%)	
Poor	16 (6.5%)	12 (8.2%)	4 (3.9%)	
**cT status**				0.657
cT2	10 (4.0%)	6 (4.1%)	4 (4.0%)	
cT3	175 (70.6%)	106 (72.6%)	69 (67.6%)	
cT4	63 (25.4%)	34 (23.3%)	29 (28.4%)	
**cN status**				0.07
cN0	38 (15.3%)	19 (13.0%)	19 (18.6%)	
cN1	101 (40.7%)	54 (37.0%)	47 (46.1%)	
cN2	109 (44.0%)	73 (50.0%)	36 (35.3%)	
**Tumour location**				0.787
< 5 cm	140 (56.5%)	85 (58.2%)	55 (53.9%)	
5–10 cm	103 (41.5%)	58 (39.7%)	45 (44.1%)	
> 10 cm	5 (2.0%)	3 (2.1%)	2 (2.0%)	
**CEA level**				0.731
Normal	143 (57.7%)	86 (58.9%)	57 (55.9%)	
Abnormal	105 (42.3%)	60 (41.1%)	45 (44.1%)	
**CA199 level**				0.550
Normal	196 (79.0%)	113 (77.4%)	83 (81.4%)	
Abnormal	52 (21.0%)	33 (22.6%)	19 (18.6%)	
**Chemotherapeutic regimen**				0.001
5-fluorouracil	35 (14.1%)	16 (11.0%)	19 (18.6%)	
Capecitabine	33 (13.3%)	10 (6.8%)	23 (22.5%)	
FOLFOX	150 (60.5%)	104 (71.2%)	46 (45.1%)	
Other regimens	30 (12.1%)	16 (11.0%)	14 (81.4%)	
**Time interval between nCRT and surgery**				0.046
< 7 weeks	13 (5.3%)	4 (2.8%)	9 (8.8%)	
7–10 weeks	91 (36.8%)	50 (34.5%)	41 (40.2%)	
> 10 weeks	143 (57.9%)	91 (62.8%)	52 (51.0%)	
**Neoadjuvant radiotherapy dose**				0.012
< 45 Gy	20 (8.1%)	6 (4.1%)	14 (13.7%)	
≥ 45 Gy	228 (91.9%)	140 (95.9%)	88 (86.3%)	

### Evaluation of the TSR

**Fig. 2:**
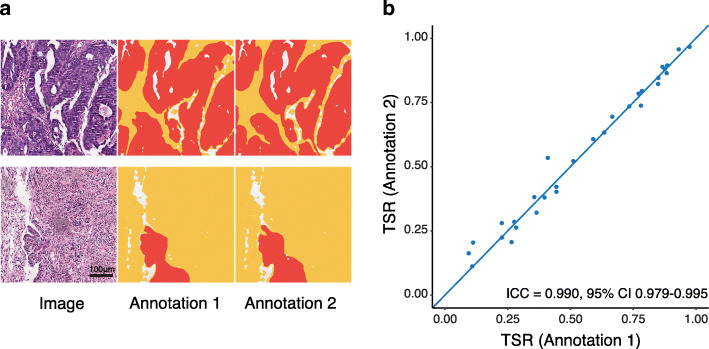
Consistency analysis of inter-observer variability of the annotations of two pathologists. (**a**) Examples of two pathologists’ annotations. (**b**) Excellent concordance was observed between the two annotations

**Fig. 3:**
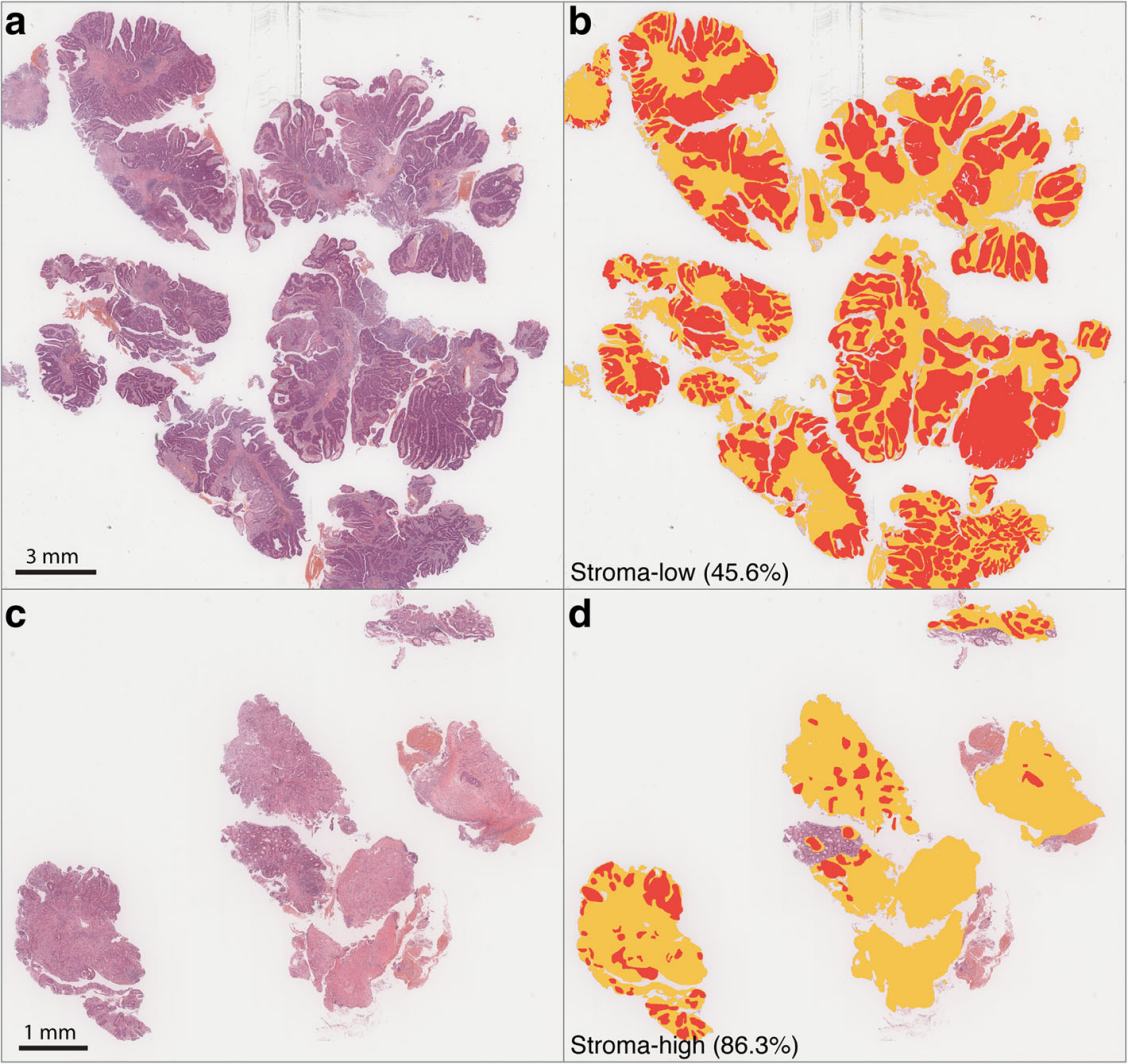
Representative images of stroma-low and stroma-rich tumour biopsies. (**a**, **c**) HE-stained biopsy sections of LARC with low TSR and high TSR, respectively. (**b**, **d**) The same regions segmented by a semi-automatic method for calculation of the TSR. HE, haematoxylin and eosin; LARC, locally advanced rectal cancer; TSR, tumour-stroma ratio

We analysed a total of 248 HE-stained biopsy sections from 248 patients. Fig. 2a shows examples of the annotations from two pathologists. Excellent concordance was observed between the two annotations (ICC 0.990, 95% CI 0.979–0.995, Fig. 2b). Representative images of stroma-low and stroma-high WSIs and the semi-automatic segmentation results are depicted in Fig. 3.

Using 56.3 and 72.8% as the cutoffs, the continuous TSR was divided into three categories. Eighty-three patients (33.5%) were considered as stroma-low, 84 patients (33.9%) as stroma-intermediate, and 81 patients (32.7%) as stroma-high. When the TSR was separated into two groups using 50% as the cutoff, a total of 193 (77.8%) patients were categorized as stroma-high and 55 (22.2%) as stroma-low.

### The TSR and other predictive factors for treatment response

**Fig. 4:**
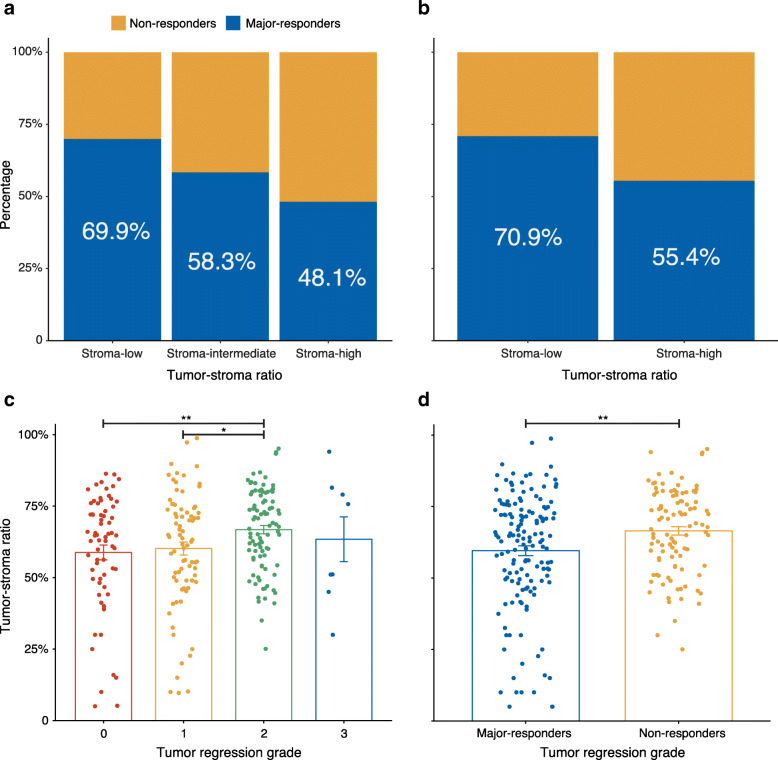
TSR can discriminate patients as major responders (TRG 0–1) from non-responders (TRG 2–3). (**a**) The distribution of major responders (in blue) and non-responders (in yellow) within the 3-category TSR. (**b**) The distribution of major responders (in blue) and non-responders (in yellow) within two stroma categories. (**c**) The distribution of TRG categories versus the continuous TSR. (**d**) The distribution of major responders and non-responders versus the continuous TSR. TRG, tumour regression grade; TSR, tumour-stroma ratio

Figure. 4a-b shows that the TSR can discriminate major-responders patients from non-responders, either dichotomous or three-class classification (*P* < 0.05, *χ2* test). Additionally, the differences were more apparent among the three-class classification. In the stroma-low group, 58 (69.9%) patients were major-responders and only 39 (48.1%) patients were considered non-responders in the stroma-high group (*P* = 0.018). The distribution of TRG categories versus continuous TSR is shown in Fig. 4c. With the increase in TRG level, the mean value of TSR also increased (except TRG 3). The distribution of major-responders and non-responders versus the continuous TSR is shown in Fig. 4d.

As illustrated in Table 1, there were no statistically significant differences between the major-responder and non-responder groups in most of the clinicopathological characteristics concerning age, sex, histopathological type, differentiation grade, clinical T/N status, tumour location, CEA, and CA199 level. By univariate analysis, the time interval to surgery following nCRT > 10 weeks was associated with a greater chance of a response to nCRT (odds ratio [OR] 3.94, 95% CI 1.16–13.4, *P* = 0.028), but not by multivariate analysis. By multivariate analysis, neoadjuvant radiotherapy doses ≥45 Gy (adjusted OR 3.71, 95% CI 1.38-10.0, *P* = 0.002) and receiving the FOLFOX regimen (adjusted OR 2.68, 95% CI 1.27–5.68, *P* = 0.007) were determined to be independent predictors of a good response to nCRT. A high TSR was independently associated with a greater chance of no response to nCRT (adjusted OR 0.40, 95% CI 0.21-0.76, *P* = 0.002, Table 2).

**Table 2:** Uni- and multivariable analyses with the logistic regression

	Univariable analysis		Multivariable analysis	
	OR (95% CI)	***P***	AOR (95% CI)	***P***
**Age (year, mean ± SD)**	0.99 (0.97–1.01)	0.505		
**Sex**				
Male	Ref			
Female	0.81 (0.46–1.42)	0.458		
**Histopathological type**				
Adenocarcinoma	Ref			
Mucinous adenocarcinoma	1.42 (0.42–4.85)	0.575		
**Differentiation grade**				
Well or moderate	Ref			
Poor	2.19 (0.69–7.01)	0.185		
**cT status**				
cT2	Ref			
cT3	1.02 (0.28–3.76)	0.971		
cT4	0.78 (0.20–3.04)	0.722		
**cN status**				
cN0	Ref			
cN1	1.15 (0.54–2.42)	0.715		
cN2	2.03 (0.96–4.30)	0.065		
**Tumour location**				
< 5 cm	Ref			
5–10 cm	0.83 (0.50–1.40)	0.491		
> 10 cm	0.97 (0.16–6.00)	0.974		
**CEA level**				
Normal	Ref			
Abnormal	0.88 (0.53–1.47)	0.636		
**CA199 level**				
Normal	Ref			
Abnormal	1.28 (0.68–2.40)	0.450		
**Time interval between nCRT and surgery**				
< 7 weeks	Ref			
7–10 weeks	2.74 (0.79–9.56)	0.113		
> 10 weeks	3.94 (1.16–13.4)	0.028		
**Chemotherapeutic regimen**				
5-fluorouracil	Ref		Ref	
Capecitabine	0.52 (0.19–1.40)	0.194	0.45 (0.16–1.28)	0.134
FOLFOX	2.68 (1.27–5.68)	0.010	2.93 (1.33–6.46)	0.007
Other regimens	1.36 (0.51–3.61)	0.541	1.53 (0.55–4.28)	0.415
**Neoadjuvant radiotherapy dose**				
< 45 Gy	Ref		Ref	
≥ 45 Gy	3.71 (1.38–10.0)	0.006	5.25 (1.84–14.9)	0.002
**TSR (three categery)**				
Low	Ref		Ref	
Intermediate	0.60 (0.32–1.14)	0.121	0.45 (0.22–0.90)	0.025
High	0.40 (0.21–0.76)	0.005	0.32 (0.16–0.65)	0.002

*Abbreviation*: *TSR* tumour-stroma ratio

## Discussion

In this study, we evaluated the TSR by a semi-automatic quantification method from preoperative biopsy specimens is the hope of selecting patients with LARC who would or would not benefit from nCRT. Patients with high amounts of stroma have a significantly superior chance of not responding to nCRT (TRG 2–3) compared with patients with low stroma.

Tumour regression grade is an important approach to assess the response to nCRT in LARC patients [22], which is assessed by a histopathologist largely based on the proportion of residual tumour cells and fibrosis. It is possible that tumours with a high stromal content may be misclassified as showing a greater degree of regression than the reality due to the desmoplastic stromal reaction. However, it is notable that the amount of stroma is inversely correlated with the degree of tumour regression in our research, which is contrary to the above inference. This further demonstrates that there is an association between TSR and tumour regression grade. The more stroma there is, the lower the degree of tumour regression, which indicates a poorer prognosis. This result is consistent with other studies showing that a higher TSR is associated with a worse prognosis [23, 24].

Most of the previous literature has studied the TSR based on surgical specimens [13, 14, 25, 26]. Our study evaluated TSR on preoperative diagnostic biopsy sections of rectal cancer. Likewise, in digestive tract tumours, the results of oesophageal cancer research are consistent with our research [7, 15]. They found that the TSR can predict the response to nCRT in oesophageal cancer patients, but they assessed the TSR in artificially selected areas by visually subjective semi-quantitative scoring (10, 20%, ..., 100%). Our study adopted a full quantification method on WSIs in LARC patients. Compared with the results from Pelt et al. (Kappa = 0.73) [7], our method achieved a higher degree of agreement for TSR evaluation (ICC = 0.99). Furthermore, the multivariate analysis uncovered that only the TSR, neoadjuvant radiotherapy dose and FOLFOX regimen were independent predictive factors. However, the two latter variables are markers in treatment, and only TSR is a pre-treatment predictor. From the perspective of changing treatment schemes, preoperative assessment of the TSR is more likely to guide adaptive treatment planning for LARC patients. Owing to the differences in the areas evaluated for TSR in biopsy specimens and surgical specimens, the use of 50% as the cutoff which has been reported in the preceding literature may not be applicable to biopsy specimens. The tertile approach used in our study may be more appropriate for evaluation of the TSR of biopsy specimens.

There are also studies that use preoperative biopsy HE histology image analysis to predict the nCRT response of LARC patients. For example, Zhang et al. obtained quantitative features of preoperative biopsy HE-stained histology slides through machine learning and investigated its capability in predicting the treatment response of patients to nCRT [27]. Nevertheless, these signatures lack biological interpretability and are not easily translated into routine pathologic assessments. In our study, the relation between the TSR and the response to nCRT may be explained pathophysiologically. Extensive research has shown that the stroma plays a major role in the induction of chemoradiotherapy resistance [28]. For instance, chemoradiotherapy-induced damage in the tumour environment induces stromal cell stress, which in turn secretes additional factors that accelerate cancer cell proliferation, survival, invasion, and metastasis [29]. Therefore, our outcome that patients with high amounts of stroma are more likely to be related to poor response to nCRT has good interpretability.

Our findings did not show a significant difference in pathological complete response (pCR: TRG 0 vs. TGR 1–3) between the stroma-high group and the stroma-low group, but we found a significant difference between major-responders and non-responders. Additionally, as the tumour regression grade increased, the stroma content showed an upward trend (TRG 3 was not included due to the small number of cases). The lack of a pCR difference might be due to the small number of TRG 0 cases (27.0%). LARC patients with pCR may choose a “watch & wait” policy [30]. However, the main objective of this article was to select LARC patients who could benefit from nCRT, so it may be more meaningful to study patients who obtain good responses.

To our knowledge, no published studies to date have explored the relationship between the TSR and the response after nCRT in LARC patients. Unlike other studies [26], this is the first to assess the relative amounts of tumour and stroma by a semi-automatic method from preoperative diagnostic biopsy sections. The present study shows that patients with more stromata are less likely to benefit from nCRT. This might indicate that patients with high amounts of stroma that will probably not respond to nCRT, should adjust their therapeutic strategy. For instance, these tumours could receive therapies targeting stromal elements in combination with cytotoxic drugs. Another alternative is that they might be able to proceed directly to radical surgery without the use of neoadjuvant chemoradiotherapy, thereby avoiding exposure to the side effects of chemoradiation treatment. However, these conclusions need to be further validated in long-term, large-sample prospective studies.

Our study had several limitations. First, this was a retrospective study from a single center. The number of cases was too small to conduct validation. Further multicenter and prospective studies in a large cohort are needed in the future before implementing the TSR in routine clinical practice. Second, the TRG was used as the endpoint outcome as the patient’s DFS and OS information was not available. In addition, in the current study, we used a semi-automatic method to evaluate the TSR, which retained some subjective deviations and required manual annotation. However, based on the images annotated in this paper, we will develop an artificial intelligence based method to perform pixel-level tumour-stroma segmentation in future research.

In conclusion, this study introduced a semi-automatic method to quantify the TSR and proved that it is an important parameter to predict the response of patients with LARC who underwent nCRT. This might indicate that the TSR may potentially be a powerful tool to identify patients who will benefit from nCRT, thereby guiding the clinicians to select suitable management strategies and adjusting the treatment methods in a timely manner to minimize side effects and medical expenses.

## Supplementary Information

**Additional file 1: Supplementary Methods.**

## Data Availability

The datasets used and/or analyzed during the current study are not publicly available due to personal information involved but are available from the corresponding author on reasonable request.

## Abbreviations

CRC: Colorectal cancer
nCRT: Neoadjuvant chemoradiotherapy
LARC: Locally advanced rectal cancer
OS: Overall survival
TSR: Tumour-stroma ratio
WSI: Whole-slide image
SYSU6: Sixth Affiliated Hospital of Sun Yat-sen University
HE: Haematoxylin and eosin
ICC: Intra-class correlation coefficient
CI: Confidence interval
TRG: Tumour regression grade
pCR: Pathological complete response
