# Supplementary material for: The value of the tumour-stroma ratio for predicting neoadjuvant chemoradiotherapy response in locally advanced rectal cancer: a case control study
Source: BMC Cancer. 2021 Jun 25;21:729. doi: 10.1186/s12885-021-08516-x (PMC8235870; doi:10.1186/s12885-021-08516-x)
Supplement: Supplementary file 1 — Additional file 1: Supplementary Methods. [file 12885_2021_8516_MOESM1_ESM.doc]

**Supplementary Materials**

**Title:** The value of the tumour-stroma ratio for predicting neoadjuvant chemoradiotherapy response in locally advanced rectal cancer: A case control study

**Authors:** Yanting Liang^1#^, Yaxi Zhu^2#^, Huan Lin^3,4#^, Shenyan Zhang^2#^, Suyun Li^3,4^, Yanqi Huang^4,5^, Chen Liu^4,5^, Jinrong Qu^6^, Changhong Liang^4^, Ke Zhao^3,4*^, Zhenhui Li^7*^, Zaiyi Liu^4*^

**Affiliations:**
^1^Guangdong Cardiovascular Institute, Guangdong Provincial People's Hospital, Guangdong Academy of Medical Sciences, Guangzhou, China
^2^Department of Pathology, The Sixth Affiliated Hospital of Sun Yat-sen University, Guangzhou, China
^3^School of Medicine, South China University of Technology, Guangzhou, China
^4^Department of Radiology, Guangdong Provincial People's Hospital, Guangdong Academy of Medical Sciences, Guangzhou, China
^5^The Second School of Clinical Medicine, Southern Medical University, Guangzhou, China
^6^Department of Radiology, The Affiliated Cancer Hospital of Zhengzhou University & Henan Cancer Hospital, Zhengzhou, China
^7^Department of Radiology, The Third Affiliated Hospital of Kunming Medical University, Yunnan Cancer Hospital, Yunnan Cancer Center, Kunming, China

^#^These authors contributed equally to this work.

**^*^Corresponding authors:**

Zaiyi Liu
liuzaiyi@gdph.org.cn
Department of Radiology, Guangdong Provincial People's Hospital, Guangdong Academy of Medical Sciences, 106 Zhongshan Er Road, Guangzhou, 510080, China

Or Zhenhui Li
lizhenhui621@qq.com
Department of Radiology, The Third Affiliated Hospital of Kunming Medical University, Yunnan Cancer Hospital, Yunnan Cancer Center, Kunming, 650118, China

Or Ke Zhao
ksw2024@163.com
Department of Radiology, Guangdong Provincial People's Hospital, Guangdong Academy of Medical Sciences, 106 Zhongshan Er Road, Guangzhou, 510080, China

**Supplementary Methods**

**Inclusion criteria：**

For locally advanced rectal cancer (LARC) patients, those who meet all of the following inclusion criteria will be enrolled into this study:

(a) Primary rectal adenocarcinoma confirmed via biopsy

(b) Patients defined as LARC (cT3–4/N0–2/ M0) based on pre-treatment computed tomography of the chest and abdomen and pelvis magnetic resonance imaging, according to the 8th edition of the AJCC Staging Manual

(c) A tumor that was located within 15 cm of the anal verge

(d) Age of ≤80 years

(e) No other treatment before the preoperative therapy

(f) Radical surgery was performed after the preoperative therapy

**Exclusion criteria:**

(a) Patients with a history of cancer

(b) Patients who did not complete preoperative radiochemotherapy
